# Characterization of SCCmec, spa types and Multi Drug Resistant of methicillin-resistant *Staphylococcus aureus* isolates among inpatients and outpatients in a referral hospital in Shiraz, Iran

**DOI:** 10.1186/s13104-019-4627-z

**Published:** 2019-09-23

**Authors:** Zahra Hashemizadeh, Nahal Hadi, Samane Mohebi, Davood Kalantar-Neyestanaki, Abdollah Bazargani

**Affiliations:** 1 Department of Bacteriology and Virology, School of Medicine, Shiraz University of Medical Science, Shiraz, Iran; 2 Department of Microbiology and Virology, School of Medicine, Kerman University of Medical Sciences, Kerman, Iran

**Keywords:** *Staphylococcus aureus*, Methicillin-resistant *S. aureus* (MRSA), SCCmec typing, Spa typing

## Abstract

**Objectives:**

Molecular typing such as spa typing is used to control and prevent *Staphylococcus aureus* widespread in hospitals and communities. Hence, the aim of this study was to find the most common types of *S. aureus* strain circulating in Shiraz via spa and SCCmec typing methods.

**Results:**

Total of 159 *S. aureus* isolates were collected from two tertiary hospitals in Shiraz. Isolates were identified by biochemical tests. Antimicrobial susceptibility tests were performed by standard disk diffusion method and then genetic analysis of bacteria was performed using SCCmec and spa typing. In this study 31.4% of the isolates were methicillin-resistant *S. aureus*. The majority of isolates were SSCmec type III. Spa type t030 was the most prominent type among MRSA strains. For the first time in Iran, spa003, t386, t1877, t314, t186, t1816, t304, t325, t345 were reported in this study. It was shown that there is a possibility that these spa types are native to this region. Our findings showed that SCCmec II, III and IV disseminate from hospital to community and vice versa. Thus, effective monitoring of MRSA in hospital and community is necessary.

## Introduction

*Staphylococcus aureus* is a major human pathogen, causing a wide range of clinical infections such as skin, soft tissue, bone, joints bloodstream, and pneumonia [1]. Also, this bacterium is one of the most common causes of nosocomial and community-acquired infections [2]. Antibiotic resistance is one of the biggest problems in public health and treatment of these infectious diseases, especially *S. aureus* infections is essential [3]. Methicillin as antimicrobial agent is known to inhibit bacterial cell-wall synthesis [4]. Methicillin resistance in staphylococci produces a penicillin binding protein 2a (PBP 2a), which is encoded by the mecA gene [4]. In 1961, emergence of methicillin-resistant *S. aureus* (MRSA) was reported in London [3].

MRSA is one of the most important nosocomial pathogens that can cause healthcare-associated infections [5, 6]. Staphylococcal cassette chromosome mec (SCCmec), with 21 to 67 kbp in size, is a mobile genetic element that causes resistance to methicillin in staphylococcus bacterial species [7]. Due to their structural organization and genetic content, SCCmec elements are classified into 11 different types (SCCmecI-XI) [8, 9]. Various techniques such as multilocus sequence typing (MLST), pulsed field gel electrophoresis (PFGE), prophage typing, biotyping, coa typing, spa typing and SCCmec have been used to determine the epidemiology of MRSA [10]. Among these epidemiological methods, spa typing is quick, less expensive and has discriminatory power [11]. The gene encoding protein A (spa) contains three functionally distinct regions: Fc binding region, X region and at C terminus [12]. The X-region contains a variable number of 24-bp repeats. Hence, spa typing, based on the sequencing of X-region was used for subtyping of *S. aureus* [13] The main goal of this study was to find the common types of *S. aureus* strains circulating in Shiraz by spa and SCCmec typing methods.

## Main text

### Material and method

#### Sample collection

The present study was conducted on 159 *S. aureus* isolates collected from two tertiary hospitals (Namazi and Faghihi), Shiraz from December 2017 to September 2018. The strains were isolated from clinical samples including pus, wound, blood, sputum and cerebrospinal fluid. All isolates were cultured on the sheep blood agar and were identified by catalase, tube coagulase, mannitol fermentation and DNase tests [14].

#### Antimicrobial susceptibility

Antimicrobial susceptibility tests were performed, using standard disk diffusion method on Mueller–Hinton agar (Merck, Germany) according to CLSI 2018 standards guidelines (Clinical and Laboratory Standards Institute). We used antibiotic disks of penicillin (5 μg), ceftaroline (30 μg), clindamycin (2 μg), trimethoprim–sulfamethoxazole (2.5 μg), gentamicin (10 μg), erythromycin (15 μg), azithromycin (15 μg), ciprofloxacin (5 μg), rifampicin (5 μg), chloramphenicol (30 μg), norfloxacin (10 μg), and nitrofurantoin (300 μg) (Mast, UK). The minimum inhibitory concentration (MIC) for vancomycin were determined by E-test (AB BIODISK, Sweden) method [15]. *S. aureus* ATCC 25923 was used as the control strain.

#### Screening for methicillin resistance

Resistance cefoxitin (FOX, 30 µg) was detected by growth on Mueller–Hinton agar (Merck, Germany) according to the guidelines of Clinical and Laboratory Standard Institute (CLSI) for MRSA strains detection [15].

#### MRSA screening

DNA all cefoxitin-resistant *S. aureus* isolates was extracted by a kit Exgene Clinic SV (GeneALL, Seoul, Korea). Then isolates were confirmed for mecA gene by PCR [16].

#### SCCmec typing

Different types of SCCmec were carried out by the method described by Oliveira et al. [17]. Different types of SCCmec were studied by multiplex-PCR assay with specific primers for SCCmec types I, II, III, IV.

Amplification of SCCmec genes were subjected to final volume of 25 mL containing 12.5 mL Master mix (Amplicon, Denmark), 0.2 mL of each primer with concentration of 10 pmol/mL, and 2 mL of DNA template top up to 25 mL. The PCR protocol comprised of an initial denaturation step at 94 °C for 5 min, followed by 35 cycles of denaturation at 95 °C for 60 s, annealing 55–59 °C for 60 s, extension at 72 °C for 1 min, and was followed by a final cycle of extension for 5 min at 72 °C. PCR products were detected by electrophoresis, using agarose 2% and stained with SYBER DNA safe stain, and then visualized under UV light.

#### Spa typing

The X region of the spa gene was amplified, using spa-1113f (5-TAA AGA CGA TCC TTC GGT GAG C-3) and spa-1514r (5-CAG CAG TAGTGC CGT TTG CTT-3) primers [18]. Amplification was carried out in a condition similar to what was mentioned in SCCmec typing with the annealing temperature (55 °C). PCR products were sequenced (Macrogen, Co, Korea) and then data were analyzed by (http://www.spaserver.ridom.de).

#### Statistical analysis

Statistical analysis was performed by Chi-square test (SPSS v.22) statistics software. A significant difference was considered at P value of < 0.05.

### Results

Total of 159 *S. aureus* isolates were collected from different clinical samples including Skin (35.8%), Blood (28.9%), Wound (11.9%), Fluid (7.5%), Nasal (6.9%), Sputum (5%), Auxiliary (1.9%), Eye (1.3%), and Abscess (0.6%) were collected from Faghihi and Nemazee Hospitals in Shiraz. The 159 isolates were collected from 54.1% male and 45.9% female patients. In the present study, 109 (68.5%) isolates were MSSA and 50 (31.4%) were MRSA according to the disc diffusion and the *mec*A analysis by PCR. The rate of MRSA isolates was 27.9% and 39.5% in Faghihi and Nemazee hospitals, respectively (Table 1).

**Table 1:** Distribution of gender, sample, ward, sccmec, spa types and pattern of antibiotics in MRSA isolates

Number strain	Gender	Hospital	Sample	Ward	Spa typing	*Sccmec* type	Pattern resistance of antibiotics
1	F	Faghihi	Skin	Dermatology	t030	I	P, E, AZT, CC, FM
4	M	Faghihi	Skin	Dermatology	t021	III	P, CIP, E, AZT, SXT, GM, NOR, CEF
6	M	Faghihi	Nasal	OUT	t030	IIIA	SXT
9	F	Faghihi	Fluid	Internal	t030	I	P, CIP, AZT, SXT, CC, GM, NOR, CEF
12	F	Faghihi	Nasal	OUT	t386	IIIA	P, CIP, E, AZT, SXT, CC, GM, NOR, CEF
16	M	Faghihi	Wound	Dermatology	t314	II	P, CIP, GM, FM
23	F	Faghihi	Skin	OUT	t386	IV	P, CIP, E, AZT, SXT
27	F	Nemazee	Blood	Emergency	t030	–	P, GM
33	M	Faghihi	Wound	Internal	t030	IV	P
38	F	Faghihi	Skin	Dermatology	t021	III	CIP, SXT, NOR
39	F	Faghihi	Skin	Dermatology	t030	II	P, CIP, E, AZT, CC, GM, NOR, CEF
40	F	Faghihi	Blood	Internal	t 386	–	P, CIP, E, AZT, NOR, CEF
41	F	Faghihi	Skin	Dermatology	t021	IA	CIP, E, AZT, CC, NOR, FM
43	M	Faghihi	Skin	Dermatology	t037	III	P, CIP, E, AZT, SXT, CC, NOR, FM, RP, CEF
44	M	Faghihi	Sputum	ICU	t021	I	SXT
46	M	Faghihi	Skin	Dermatology	t 186	IV	P, E, AZT
48	F	Faghihi	Skin	OUT	t1877	III	P
49	F	Faghihi	Skin	Dermatology	t314	IV	P, E, AZT, CC, NOR
50	F	Faghihi	Skin	Dermatology	t021	IV	P, E, AZT
65	F	Faghihi	Skin	Dermatology	t345	I	P, CIP, E, AZT, SXT, CC, GM, NOR, CEF
70	M	Faghihi	Skin	Dermatology	t 1877	III	P, CIP, E, AZT, SXT, CC, GM, NOR, CEF
73	F	Faghihi	Skin	OUT	t1877	IV	P, E, AZT, SXT, CC, NOR, CEF
75	M	Faghihi	Skin	Dermatology	t790	IV	P
79	M	Faghihi	Blood	Internal	t1877	III	P, CIP, E, AZT, SXT, CC, NOR, CEF
92	M	Faghihi	Wound	Dermatology	t037	IA	P, CIP, E, AZT, CC, NOR
96	F	Faghihi	Wound	OUT	t790	IV	P, E, AZT, CC, CEF
97	M	Nemazee	Skin	OUT	t386	II	P, E, AZT, CEF
99	F	Nemazee	Blood	Internal	t386	–	P, E, AZT, CEF
100	M	Nemazee	Blood	Emergency	t030	III	P, CIP, E, AZT, CC, GM, NOR, FM, RP, CEF
103	M	Nemazee	Blood	Emergency	t030	III	P, CIP, E, AZT, CC, GM, NOR, RP, CEF
106	F	Faghihi	Nasal	OUT	t1877	–	P
108	F	Nemazee	Abses	Emergency	t021	III	P, CIP, E, AZT, SXT, NOR
109	M	Nemazee	Blood	Emergency	t325	IV	P
110	F	Nemazee	Blood	OUT	t325	IV	P, CEF
117	M	Faghihi	Skin	Dermatology	t021	IV	P, CIP, E, AZT, SXT, NOR
118	F	Faghihi	Skin	Dermatology	t037	IIIA	P, CIP, E, AZT, SXT, GM, NOR, CEF
121	F	Nemazee	Blood	Emergency	t030	II	P
122	F	Nemazee	Wound	OUT	t1816	IV	P, E, AZT, CEF
124	M	Nemazee	eye	ICU	t304	III	P, CEF
125	M	Faghihi	Wound	Internal	t021	III	P, CIP, SXT, NOR
126	M	Faghihi	Wound	Internal	t021	III	P, CIP, SXT, NOR
127	M	Faghihi	Skin	Dermatology	t003	II	PEN
128	F	Nemazee	Sputum	ICU	t1877	IV	P, E, AZT
150	M	Nemazee	Sputum	ICU	t021	III	P, E, AZT
151	M	Nemazee	Sputum	ICU	t030	IIIA	P, CIP, E, AZT, SXT, CC, NOR, CEF
154	F	Nemazee	Sputum	ICU	t037	III	P, CIP, E, AZT, CC, GM, NOR, CEF
155	M	Nemazee	Wound	OUT	t018	–	P, CIP, E, AZT, CC, , GM, NOR, CEF
156	F	Nemazee	Nasal	Emergency	t081	III	P
157	F	Nemazee	Blood	Internal	t030	II	P, CIP, E, AZT, SXT, CC, GM, FM, RP, CEF
159	M	Nemazee	Nasal	OUT	t030	III	P, CIP, E, AZT, CC, GM, NOR, CEF

*F* female, *M* male, *OUT* outpatient, *P penicillin*, *C* chloramphenicol, *CIP* ciprofloxacin, *E* erythromycin, *AZT* azithromycin, *SXT* trimethoprim–sulfamethoxazole, *CC* clindamycin, *GM* gentamicin, *NOR* norfloxacin, *FM* nitrofurantoin, *RP* rifampicin, *CEF* ceftaroline

#### Antibiotic resistance

The resistance patterns were observed amongst the isolates: penicillin (78.6%), erythromycin (45.9%), azithromycin (45.3%), norfloxacine (28.3%), trimethoprim–sulfamehoxazole (27%), ciprofloxacin (27%), clindamycin (22%), cefaroline (23.3%), gentamicin (10.7%), nitrofurantoin (4.4%), chloramphenicol (3.1%), and rifampicin (3.1%). All the isolates were susceptible to vancomycin according to *E*-test method. In this study, the rate of multidrug resistance (MDR) among isolates was 31.4% of which 24 and 22 resistance profiles were detected among MSAA and MRSA isolates, respectively. The antibiotics resistance results between MSAA and MRSA are presented in Table 2. There was a statistical correlation between the rate of antibiotic resistance and MSAA and MRSA, except for trimethoprim–sulfamethoxazole and chloramphenicol (*P *> 0.05). The highest rate of MDR was isolated from skin, blood and wound, as well as from dermatology, internal and outpatient wards.

**Table 2:** Antibiotic resistance profiles of 159 isolates of MRSA and MSSA

Isolates (N)	Resistance of antibiotics N (%)											
	P	C	CIP	E	AZT	SXT	CC	GM	NOR	FM	RP	CEF
MRSA (50)	46 (92)	0 (0)	27 (54)	33 (66)	33 (66)	19 (38)	21 (42)	14 (28)	25 (50)	6 (12)	4 (8)	24 (48)
MSSA (109)	79 (72.4)	5 (4.5)	17 (15.5)	41 (37.6)	40 (36.6)	26 (23.8)	17 (15.5)	3 (2.7)	20 (18.3)	2 (1.8)	1 (0.9)	13 (11.9)
P value	0.003	0.1	0.0001	0.002	0.001	0.07	0.001	0.0001	0.0001	0.02	0.03	0.0001

*P penicillin, C* chloramphenicol, *CIP* ciprofloxacin, *E* erythromycin, *AZT* azithromycin, *SXT* trimethoprim–sulfamethoxazole, *CC* clindamycin, *GM* gentamicin, *NOR* norfloxacin, *FM* nitrofurantoin, *RP* rifampicin, *CEF* ceftaroline

#### SCCmec and spa typing

Of the 50 MRSA strains, 4 (8%) harbored SCCmec type I, 2 (4%) SCCmec type IA, 6 (12%) SCCmec type II, 14 (28%) SCCmec type III, 2 (4%) SCCmec type IIIA, 13 (26%) SCCmec type IV and 6 (12%) of them were nontypeable by Oliveira method. The most prevalent SCCmec MRSA was SCCmec type III. In the outpatient isolates prevalence of SCCmec type IV was 13 (26%). Ultimately, among the 50 MRSA, typing was performed using spa typing method. Sequencing of *spa* gene revealed 15 different spa types, and spa type t030 (n = 12; 24%) was the most notable type among MRSA strains, followed by types t021 (n = 10; 20%), t386 (n = 5;10%), t1877 (n = 6;12%), t037 (n = 4;8%), t314 (n = 2;4%), t0790 (n = 2;4%), t325 (n = 2;4%), t018, t081, t186, t1816, t304, t003, t345 (n = 1, 2%). Distribution of samples, hospital wards, spa typing, SCCmec types and pattern of antibiotic among MRSA isolates are show in Table 1.

### Discussion

The widespread of MDR *S. aureus* is becoming a serious challenge in public health. Recently, therapeutic options against MDR *S. aureus* have become limited, causing morbidity and mortality in hospitalized patients [19]. Inappropriate use of antibiotics in hospitals and communities has resulted in an increasing resistance to various antibiotics, especially beta lactam antibiotics [20].

In this study, the maximum resistance of MRSA isolates was to beta lactam antibiotics such as penicillin (92%), followed by erythromycin, azithromycin (66%) and ciprofloxacin (54%). The rate of resistance to antibiotics was higher in MRSA isolates in comparison with MSSA isolates. Rate of resistance to chloramphenicol, rifampicin, nitrofurantoin and gentamicin was lower than the other antibiotics in MRSA and MSSA. These were in line with other studies from Iran [20], which can be due to wide use of these antibiotics to treat various infections in hospitals. In the present study, high rate of resistance to clindamycin, erythromycin, ciprofloxacin antibiotics was consistent with the data from a previous study in Iran [18]. In this study, out of 31 isolates of *S. aurous* isolated from outpatients, 12 and 19 isolates were MRSA and MSSA, respectively. At present, resistance to different antibiotics is on the rise, which can lead to inappropriate use of antibiotics in communities, hospitals and agricultural industry. Hence, infective infection control policies in hospitals can be the cause of increased antibiotic resistance in the communities and hospitals [21].

In our study, vancomycin antibiotic was a highly effective against MRSA isolates, which was consistence with other studies in Iran [11, 20, 22].

MRSA isolates are one of the most common causes of threat to public health [23]. In the current study, the prevalence of MRSA isolates was 31.4%, which was lower than what was reported in other studies in Iran [24–27]. The differences in the distribution of this gene could support the different infection control policies, studied population, the diversity types of clinical isolates, inappropriate use of antibiotics, and prescribing certain antibiotics in different geographic areas.

SCCmec typing provides useful information about the resistance of genes to methicillin, and to identify the origin of strains [28, 29]. In our study, SCCmec typeIII was the most common type, which was similar to other reports in Iran and other countries [11, 18, 22, 26, 27, 30, 31]. However, in a study by Havaie et al. SCCmec type IV was the most common SCCmec type [32]. In the current study, frequency of SCCmec type IV in comparison to other SCCmec type was high (26%), which might be due to their small size that can spread among *S. aureus* isolates collected from hospitals and communities [13]. Rate of SCCmec type IV among outpatients (10%) and hospitals (16%) was reported to be the highest amongst the samples isolated from the skin. The results of SCCmec typing revealed that types II, III, IV were the most common types in outpatients. These results show that MRSA isolates could be transmitted from hospitals to communities and vice versa, while type IV belongs to CA-MRSA.

Among the MRSA isolates, 15 different spa types were revealed, and Spa type t030 was the most frequent type (n = 12, 24%) followed by Spa type t021 (n = 10, 20%). In Iran, different types of spa were reported [11, 18, 20, 32] and the most common types are as follows: t021, t037, t701, t790 related to MRSA isolates, which is similar to other studies in Iran [18, 20], but in our study, spa types t030, t386, t314, t1877, t325, t345, t304, t003, t81 and t018 were detected. In this study, spa type t037 belonged to SCCmec type III, which is in agreement with Darban et al. study from Iran [11]. Spa type 790 is considered as SCCmec type IV and other spa types belonged to different SCCmec types, which were in line with other studies, for example spa type t030 belong to SCCmec types of I, II, III, IV [18]. According to previous data, t030 spa type is commonly recognized as MRSA, which was similar to our study [11, 18].

In different countries, various spa types among clinical isolates were reported. In Europe, spa t032, in Asia, spa type t030, in America, t008, in Africa, t037 and in Australia, t202 were the most common types [2].

As far as we know, in the present study, spa type t003, t386, t1877, t314, t186, t1816, t304, t325, t345 is the first to be reported in Iran. Spa type t003 was reported in European and American countries, and its presence in Iran might be due to the spread of this spa from one continent to another.

### Conclusion

High rate of MDR among MRSA isolates requires a new policy in order to control infection. In this study, we showed that the diversity of spa types in MRSA isolates was high and spa type t030 was found to be the most frequent. Also, for the first time in Iran spa type’s spa003, t386, t1877, t314, t186, t1816, t304, t325, t345 were reported.

## Limitations

A limitation in this study was that we did not carry out typing of MSSA isolates, due to financial constraints.

## Data Availability

The data that support the findings of this study are available. Anyone interested can get upon reasonable request from corresponding author.

## Abbreviations

MRSA: methicillin-resistant *S. aureus*
PBP 2a: penicillin binding protein 2a
SCCmec: staphylococcal cassette chromosome mec
MLST: multilocus sequence typing
PFGE: pulsed field gel electrophoresis:
MDR: Multi Drug Resistant
CA-MRSA: community-acquire
CLSI: Clinical and Laboratory Standard Institute
